# Effect of One-Year Growth Hormone Therapy on Cardiometabolic Risk Factors in Boys with Obesity

**DOI:** 10.1155/2020/2308124

**Published:** 2020-02-19

**Authors:** Jing Wu, Fei Zhao, Yuan Zhang, Jiang Xue, Jiangying Kuang, Zhi Jin, Taie Zhang, Chunjie Jiang, Dingding Wang, Shuang Liang

**Affiliations:** ^1^Department of Pharmacy, The Second Hospital of Shandong University, Jinan, Shandong, China; ^2^Department of Pediatrics, The Second Hospital of Shandong University, Jinan, Shandong, China; ^3^Center of Evidence-based Medicine, The Second Hospital of Shandong University, Jinan, Shandong, China; ^4^Department of Cardiology, The Second Hospital of Shandong University, Jinan, Shandong, China; ^5^Department of Traditional Chinese Medicine, The Second Hospital of Shandong University, Jinan, Shandong, China

## Abstract

It has been recognized that people with obesity are more likely to have low growth hormone secretion. Recent studies have also confirmed that the abnormalities of the growth hormone/insulin-like growth factor 1 axis were associated with cardiovascular complications in people with obesity. However, little is known about whether recombinant human growth hormone therapy could improve cardiovascular and metabolic risks in obese children. This study aims to evaluate the effect of one-year growth hormone therapy on obesity-related comorbidities and to assess the safety in Chinese boys with obesity. Eighteen boys with obesity were treated with recombinant human growth hormone for one year. Anthropometric measurements, endocrine testing, and cardiovascular risk markers were performed in all obese boys in baseline, and follow-up visits were performed at 3 months, 6 months, 9 months, and one year, respectively. After one year of recombinant human growth hormone treatment, the body mass index standard deviation scores decreased (*P* < 0.001) and insulin-like growth factor 1 levels increased (*P* < 0.001). GH treatment also reduced low density lipoprotein cholesterol (*P* < 0.001), total cholesterol (*P* < 0.001), triglycerides (*P*=0.042), and alanine aminotransferase (*P*=0.027) when compared with the baseline. One-year of recombinant human growth hormone treatment could improve cardiometabolic risk markers, without adverse effects on glucose homeostasis in boys with obesity.

## 1. Introduction

As obesity has become more and more popular, many people are plagued by the comorbidities associated with obesity [1]. Obesity and obesity-related comorbidities, thus, have been identified as one of the most important public health concerns in the world [1]. Therefore, more and more studies started to focus on the relevant topics related to obesity and its complications. A recent study indicated that excessive gain of gestational weight, gestational diabetes mellitus, breast feeding for a period of less than 6 months, and hypertensive disorders of pregnancy may lead to maternal and childhood obesity [2]. Skrypnik et al. [3] presented the current state of knowledge about the genetic basis of obesity complications. Early attention and detection of these risk factors may prevent the development of obesity and its complications.

Apart from obesity-related comorbidities, obesity is also associated with endocrine perturbations. In people with obesity, low growth hormone (GH) secretion has been recognized as one of their characteristics [4, 5]. The altered somatotroph of people with obesity can be reversed with weight loss, so the change of GH is relative and functional in people with obesity [4, 6]. During the last decades, several studies indicated that GH secretion was negatively associated with cardiovascular complications in obese adults [7–11]. Our previous studies also confirmed the abnormalities of the GH/insulin-like growth factor 1 (IGF-1) axis in children with obesity [12], and the reduced GH and IGF-1 secretion were independently associated with cardiovascular risk factors such as metabolic syndrome [12], hyperuricemia [13], and nonalcoholic fatty liver disease (NAFLD) [14, 15]. These findings have led to the extension of research to the clinical application of recombinant human growth hormone (rhGH) in patients with obesity to improve cardiovascular and metabolic risks. In adults with obesity, many studies have demonstrated rhGH administration reduced in visceral adiposity and improved insulin sensitivity, as well as blood lipid profiles and serum inflammatory markers [16–20]. However, although some studies focusing on the effects of rhGH on cardiovascular risk factors have largely been performed in children with GH deficiency and Prader–Willi syndrome, only few of them have estimated the effect of rhGH in obese children without structural pituitary disease.

There is only sparse evidence in the literature reporting the effects of rhGH administration on cardiometabolic risk factors based on the samples of obese children [21–23]. We reported that 6 months of GH treatment reduced body mass index standard deviation scores (BMI SDS), exerting beneficial effects on blood lipid profiles without causing deterioration on glucose homeostasis in obese children with relative GH deficiency [21]. Similar data has been produced in prepubertal obese boys that 6 months of GH treatment was able to reduce the total body fat percentage without eliciting negative effects on the glucose homeostasis [22]. Treatment with rhGH in physiologic doses for 6 months in adolescent girls with obesity was followed by a reduction in total cholesterol and soluble intercellular adhesion molecule without adversely affecting the glycemic status [23]. Although the participants in these studies differed in race, subjects, and doses of rhGH treatment, the results showed that rhGH treatment could improve cardiovascular metabolic risk factors to varying degrees without serious adverse reactions. Nevertheless, there are no long-term data on the effect of GH treatment in children with abdominal obesity. Whether longer-term rhGH replacement therapy can further exert beneficial effects on the atherosclerotic process or show more significant side effects is unknown. Hence, in the study, we observed changes in endocrine and cardiovascular metabolic factors in obese boys. Furthermore, the main objectives of our study are to evaluate the effect of one-year rhGH therapy on obesity-related comorbidities and to assess the safety in Chinese boys with obesity.

## 2. Methods and Procedures

### 2.1. Subjects

All participants and their parents gave signed informed consent, and the study was approved by the Ethics Committee of the Second Hospital of Shandong University. This study was registered with Clinical Trials.gov (ChiCTR-IPR-17011267). All procedures involving human participants were performed in accordance with the 1964 Helsinki declaration and its later amendments or comparable ethical standards.

Our inclusion criteria were obese boys aged 8–16 years with the body mass index (BMI) of each subject greater than the 95th percentile for age [24]. Exclusion criteria included (1) hypothalamic or pituitary disorders, hypothyroidism, and Cushing disease; (2) diabetes mellitus, chromosome abnormalities, or all sorts of syndromes; (3) serious infection, systemic disease, and other chronic wasting illnesses; (4) the use of drugs may influence body composition, GH secretion, or glucose and lipid metabolism; and (5) short stature or the growth velocity is less than 5 cm/year; from May 2017 to January 2018, twenty-four obese boys fulfilled the inclusion criteria and agreed to participate in the clinical trial. Three subjects withdrew for personal reasons, and three subjects were lost to follow-up and thus six subjects in total were excluded from the analysis. Finally, eighteen obese boys enrolled and completed the study. Over the same study period, eighteen healthy boys with BMI normal for age, pubertal status, height standard deviation scores (Ht SDS), attending checkups at the Department of Pediatrics, the Second Hospital of Shandong University, were consecutively recruited to the study as the control group. Written informed consent was provided by all participants and their parents. All patients have no programs of diet and exercise.

### 2.2. Protocol

Anthropometric measurements, endocrine testing, cardiovascular risk markers, and imaging parameters were performed in all boys in the baseline period. After baseline evaluation, obese boys all received rhGH therapy (Changchun JinLei SaiZeng Co.) for one year. The daily dose of GH was 0.1–0.15 IU/kg (0.033–0.05 mg/kg) administered subcutaneously before bedtime. Follow-up visits were performed at 3 months, 6 months, 9 months, and one year, respectively, and GH doses were adjusted based on weight levels at these visits. Anthropometric measurements, laboratory examinations (thyroid function, IGF-1, liver function, lipid profile, glucose metabolism indicators, hemogram, and urinalysis) were performed on all visits. Parameters were compared between obese boys and those in the control group. For obese boys, differences in parameters before and after therapy were compared.

### 2.3. Auxological Measurements

Height, weight, and pubertal stages were obtained at baseline, 3, 6, 9, and 12 months. BMI was calculated as body weight in kilograms divided by height in meters squared. Ht SDS and BMI SDS were calculated using reference values in Chinese children [24]. The pubertal developmental stage was assessed by physical examinations according to the criteria established by Tanner [25].

### 2.4. Endocrine Testing and Cardiovascular Metabolism Index

Fasting serum was drawn for endocrine testing, and cardiovascular risk markers were drawn after an overnight fast. Endocrine testing includes GH, IGF-1, thyroid function, gonadal hormones, adrenal corticotropic hormone, and cortisol, followed by GH stimulation test (arginine test (0.5 g/kg, maximum 30 g) and levodopa test (10 mg/kg, maximum 0.5 g)) to measure serum GH levels at 30, 60, 90, 120, and 150 minutes. Cardiovascular metabolism indexes include alanine aminotransferase (ALT), total cholesterol (TC), high density lipoprotein cholesterol (HDL-C), low density lipoprotein cholesterol (LDL-C), triglycerides (TG), fasting blood glucose (FBG), fasting insulin, C-peptide, and glycosylated hemoglobin (HbA1C). In addition, an oral glucose tolerance test (OGTT) (1.75 g/kg, maximum 75 g) was performed in all obese boys in order to rule out diabetes mellitus. The homeostasis model assessment-insulin resistance (HOMA-IR) was applied to reflect insulin resistance and HOMA-IR = (basal insulin (*μ*IU/mL) × basal glucose (mmol/L)/22.5) [26]. The assessment of hepatitis B or C serology was performed in subjects with abnormal liver function (ALT > 50 U/L) in order to eliminate viral hepatitis.

### 2.5. Radiological Assessments

Bone age was estimated in all participants in baseline period, and one-year later, according to the method presenting by Greulich and Pyle. Magnetic Resonance Imaging scans were performed in all obese boys in baseline period.

### 2.6. Statistical Analysis

Data were analyzed using the Statistical Package for Social Sciences software for windows version 20.0 (SPSS Inc. Chicago, USA). A *P* value of less than 0.05 was considered statistically significant. All results were assessed for normality based on the method of Shapiro–Wilk test. Those variables which were found as nonparametric were log transformed using the natural log, and parametric tests were also applied. Continuous variables were expressed either as mean with standard deviation mean with standard deviation or as median with interquartile range according to their distribution. Comparison between healthy controls and obese boys was to observe the differences of endocrine and cardiovascular metabolic factors between the obese group and the control group. It was performed in baseline period using independent sample Student's *t* test or Mann–Whitney test when nonparametric distribution cannot be transformed to normal distribution (Peak stimulated GH). Categorical variables were compared by the chi-square test. Repeated measures analysis of variance (ANOVA) was used to determine any significant differences between baseline period and the period after the rhGH treatment in obese boys, and a Bonferroni adjustment was made to the alpha level for the independent *t*-tests.

## 3. Results

### 3.1. Baseline Characteristics

The baseline clinical and laboratory characteristics of obese boys and control group are described in Table 1. The two groups were matched on age, pubertal stage, and HT SDS. Significantly higher BMI SDS were observed in the obese groups (*P* < 0.001) than in the control group. Obese boys had lower peak GH based on the arginine-levodopa stimulation test (*P* < 0.001), and they also had lower IGF-1 compared with the control group (*P*=0.024). Subjects in the obese group had higher TC, LDL-C, TG, and ALT, higher levels of serum insulin, C-peptide, HOMA-IR, compared with normal-weight controls (all *P* < 0.05). It also had lower HDL-C compared with the control group (*P*=0.006). There were no significant differences in FBG.

Changes in metabolic and cardiovascular risk factors before and after one year of rhGh treatment.

Change in auxological and cardiovascular metabolic parameters of obese boys in baseline, 3 months, 6 months, 9 months, and one year is summarized in Table 2.

### 3.2. Effects of rhGH Administration on BMI SDS

**Figure 1:**
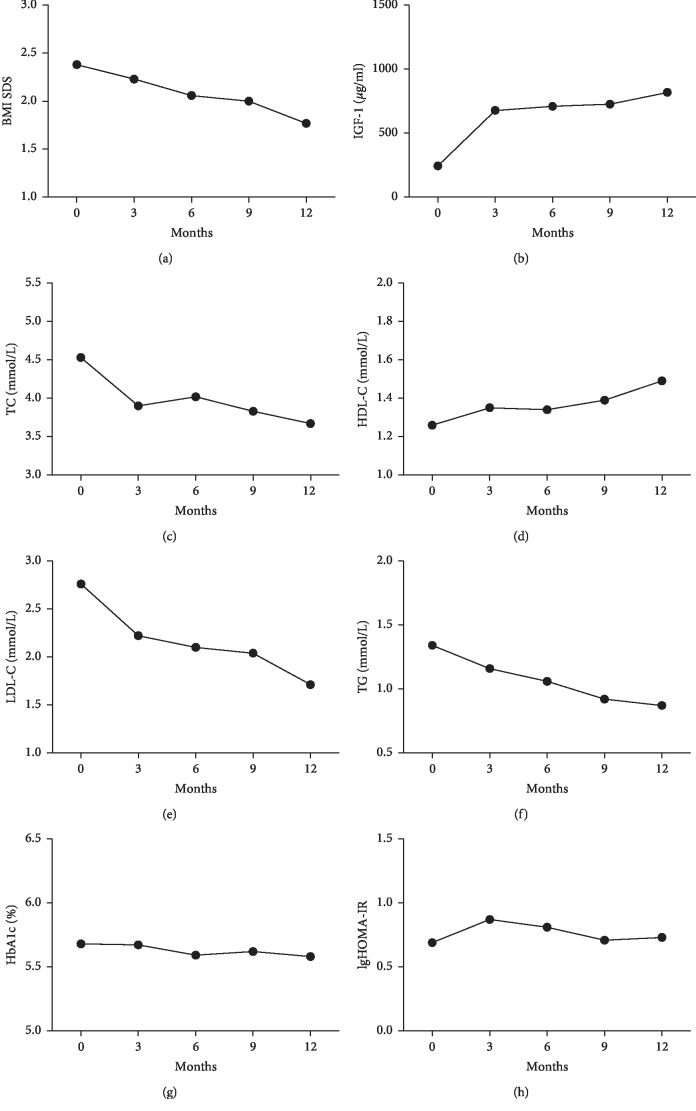
Changes in cardiometabolic risk factors and glucose metabolism before and after one year of rhGH treatment.

In obese boys, after one-year of rhGH treatment, BMI SDS decreased during GH treatment from 2.38 ± 0.39 before treatment to 2.06 ± 0.43 and 1.77 ± 0.50 after 6 and 12 months of treatment, respectively (*P*=0.006, *P* < 0.001). At the one year visit, the BMI SDS of subjects were also significantly reduced compared with the 6-month visit (*P*=0.004) (Table 2, Figure 1(a)).

### 3.3. Effects of rhGH Administration on IGF-1 Level

After one year of rhGH treatment, IGF-1 increased significantly over baseline. At the 6 month visit, there was a significant increase in IGF-1 compared with those before therapy (709.43 ± 214.72 vs. 243.44 ± 124.61 ng/mL, *P* < 0.001). At the one-year visit, IGF-1 level also significantly increased during rhGH treatment, in comparison with baseline (818.44 ± 192.96 vs. 243.44 ± 124.61 ng/mL, *P* < 0.001). However, it had no significant change when compared with the 6-month visit (818.44 ± 192.96 vs. 709.43 ± 214.72 ng/mL, *P*=0.101) (Table 2, Figure 1(b)).

### 3.4. Effects of rhGH Administration on Cardiovascular Metabolic Parameters

At the 6-month visit, LDL-C and ALT values decreased compared with those before therapy (*P* < 0.001, *P*=0.008). At the one-year visit, LDL-C, TC, TG, and ALT showed significant decreasing trend during one year of rhGH therapy in comparison with baseline (*P* < 0.001, *P* < 0.001, *P*=0.042, *P*=0.027), and LDL-C also had significant difference in comparison with the 6-month visit (*P*=0.004). In addition, after one-year of rhGH therapy, there was a rising but insignificant trend for rhGH to HDL-C when compared with baseline (1.49 ± 0.38 vs. 1.26 ± 0.24 mmol/L, *P*=0.150) (Table 2, Figures 1(c)–1(f)).

### 3.5. Effects of GH Administration on Glucose Metabolism

There was no significant difference between HbA1c before and after one-year rhGH treatment. No significant effects of GH treatment on FBG were observed after 6 month of therapy, but FBG level increased after one year of therapy compared with subjects before therapy (*P*=0.019). Three subjects experienced increases in their fasting glucose levels from <5.6 mmol/L to >6 mmol/L after one year of rhGH therapy. The three participants underwent an OGTT test, and no impaired glucose tolerance and diabetes mellitus were found in one-year visit. The HbA1c level of all participants was lower than 6%. Fasting serum insulin level showed a slightly increasing trend from 24.34 ± 13.52 *μ*IU/mL before treatment to 32.39 ± 13.07 *μ*IU/mL after the 3-month treatment, whereas the insulin decreased to 29.06 ± 15.09 *μ*IU/mL at the 6-month visit and then continued to maintain a decreasing tendency at the one-year visit. Similar changes were observed in HOMA-IR before and after one-year rhGH treatment (Table 2, Figures 1(g) and 1(h)).

### 3.6. Adverse Events

Side effects were observed in eight subjects. One subject had skin redness and swelling at the injection site, and the side effects subsided spontaneously. One subject had peripheral edema and subsided in response to a reduction in dose implemented. Three subjects had elevated FBG at the one-year visit. Two subjects had hypothyroidism during rhGH treatment, but their thyroid function returned to normal after levothyroxine treatment. One subject had leg pain, and the side effects disappeared after vitamin D and calcium supplementation. No other adverse events were reported during rhGH therapy for any of the subjects.

## 4. Discussion

In the study, we examine the effect of one year of rhGH on cardiometabolic risk factors in otherwise healthy boys with obesity. The results show that one year of GH treatment reduced the BMI SDS and ALT, raised IGF-1 levels, and improved the serum lipid pattern. Most importantly, no eliciting negative effects on the glucose homeostasis and other safety issues were raised during the one-year rhGH treatment.

Many studies demonstrated rhGH therapy may have beneficial effects on body composition in adults [16] and children [27], but the prior results related to the treatment with GH on BMI in obesity were inconsistent. Most studies using adults as samples suggested that rhGH therapy could not improve BMI in obese individuals. Nevertheless, we have previously demonstrated decreased BMI SDS following 6 months of GH administration in individuals with abdominal obesity children in comparison with the untreated control group [21]. Furthermore, in this study, we show that after one-year GH administration for obese boys, BMI SDS was significantly decreased compared with the 6 month visit and before treatment. Our results are consistent with a previous study by Kamel et al. [22], which denoted that the BMI SDS decreased after 6 months of rhGH treatment in seven prepubertal obese children. In addition, a Meta analysis which examined the efficacy of rhGH in obese adults also found that BMI decreased in response to rhGH therapy in studies of younger subjects [19]. Therefore, the age difference of participants may account for this discrepancy between these studies.

GH has a pronounced lipolytic effect, particularly in abdominal fat [28]. Previous studies have shown that administration of rhGH could improve lipid profile in patients with GHD [29] and obese adults [30]. However, data in obese adolescents were limited and conflicting. In the study, during one-year of rhGH therapy, a reduction in serum TC and LDL-C was observed in obese boys, and the reduction in LDL-C was more marked after the one-year rhGH therapy compared with the 6 month. Although this effect was not statistically significant, an increasing trend of HDL-C was also observed. Moreover, TG was also significantly reduced with the one-year GH therapy. Our previous studies also confirmed beneficial effects of GH treatment on lipid profiles after the 6-month rhGH treatment in obese children [21]. However, while Kamel et al. [22] have shown that no significant changes on the lipid profiles were observed after 6 months of rhGH treatment in prepubertal obese children, Meghan et al. [23] have reported an significant reduction in TC, an reduction trended in LDL-C, and an increasing trend in HDL-C similar to our study. The reason for this discrepancy is uncertain, but could be related to the different of subjects and the dose of GH.

Previous studies have shown that the GH replacement therapy was beneficial for NAFLD in adult GHD patients [31]. However, there were few reports on the effect of rhGH for NAFLD in otherwise healthy subjects with obesity. The rise of the levels of ALT was associated with the progression of NAFLD in obese patients [32]. Even though ALT misrepresents the entity of NAFLD, it was normally used as a valuable test to screen out pediatric NAFLD. In the study, our results demonstrated that after one year of rhGH therapy, ALT significant decreased in the obese boys. Although in our study we only performed serum ALT due to experimental conditions, the improvement of ALT may be partly verified in the potential benefits of rhGH treatment on NAFLD in obese boys.

The effect of GH on glucose metabolism is complex. Several studies have shown that acute administration of rhGH increased insulin resistance, whereas chronic GH administration might return the measures of insulin resistance to normal or even result in improvements of some studies [18, 33]. This normalization or improvement in insulin resistance might be explained by the decrease in visceral adipose induced by GH [34]. In accordance with previous studies, the response of glucose metabolism to rhGH treatment in this trial showed that the 3 months of rhGH treatment induced a temporary increase in serum insulin and HOMA-IR, but after an initial deterioration, the insulin resistance index was restored to baseline values in plasma insulin and HOMA-IR. Although FBG level was slightly higher than the baseline, the HbA1c remained within the normal ranges and none of the subjects developed diabetes mellitus and impaired glucose tolerance in obese boys after the one-year rhGH therapy. These results indicate that GH administration might have no serious negative effects on glucose homeostasis, and also the benefits of rhGH far outweigh the minimal effects on FBG.

The strength of our study is that one year of rhGH treatment achieved favorable changes with few adverse effects in obese boys. However, this study also has some limitations. First, we lacked the control group of untreated obese children to draw firm conclusions on the effect of GH treatment. One of our early studies [21] has observed the effects of short-term rhGH treatment on cardiovascular risk factors in the obese with relative GH deficiency children compared with the untreated control group. However, it is impractical for us to follow-up the controls and to repeat blood tests for one year. Second, we only considered the limited number of subjects of boys. Due to the limited sample size, gender restriction can also reduce confounding factors. Third, in the study, we did not evaluate arterial stiffness parameters, which were treated as a novel predictor of cardiovascular risk in humans [35]. Finally, it should be noted that our data merely represent a small clinical practice, and clinical recommendations cannot be made based on the results.

## 5. Conclusion

In conclusion, we have demonstrated that the one-year rhGH treatment could improve cardiometabolic risk makers, without adverse effects on glucose homeostasis, and no other serious adverse reactions occurred during the experiment. Although the reassuring findings were found in this study, further studies are required to determine the most appropriate dose and the long-term efficacy and safety of rhGH to improve cardiometabolic risk markers in obese children.

## Figures and Tables

**Table 1:** Subjects' demographic and clinical characteristics.

Variable	Obese group (*n* = 18)	Control group (*n* = 18)	*P* value
Age (year)	11.87 ± 1.43	11.53 ± 2.23	0.585
Puberty: yes	5 (18)	6 (18)	0.717
No	13 (18)	12 (18)	
HT SDS	0.21 ± 0.82	0.28 ± 1.03	0.819
BMI SDS	2.38 ± 0.39	−0.17 ± 0.77	<0.001^*∗*^
Peak-stimulated GH (*μ*g/L)	2.44 (1.49–4.24)	16.90 (11.23–18.48)	<0.001^*∗*^
IGF-1 (*μ*g/ml)	243.44 ± 124.61	337.94 ± 114.52	0.024^*∗*^
TC (mmol/L)	4.53 ± 0.61	3.90 ± 0.57	0.003^*∗*^
HDL-C (mmol/L)	1.26 ± 0.24	1.56 ± 0.36	0.006^*∗*^
LDL-C (mmol/L)	2.76 ± 0.46	2.04 ± 0.37	<0.001^*∗*^
TG (mmol/L)	1.34 ± 0.65	0.61 ± 0.23	<0.001^*∗*^
ALT (U/L)	31.24 ± 21.85	13.67 ± 3.45	<0.001^*∗*^#
Insulin (*μ*IU/mL)	24.34 ± 13.52	9.07 ± 5.29	<0.001^*∗*^#
C-peptide (*μ*g/ml)	3.39 ± 1.28	1.81 ± 0.55	0.008^*∗*^
FBG (mmol/L)	5.24 ± 0.33	5.24 ± 0.37	0.955
HOMA-IR	5.67 ± 3.12	4.39 ± 0.90	<0.001^*∗*^#

#*P* value reported for log-transformed values, but values in the table represent a back transformation to the original ^*∗*^*P* < 0.05.

**Table 2:** Change in auxological and cardiovascular metabolic parameters before, during, and after GH treatment.

Variable	Basal	3 months	6 months	9 months	1 year
BMI SDS	2.38 ± 0.39^ab^	2.23 ± 0.37	2.06 ± 0.43^c^	2.00 ± 0.42	1.77 ± 0.50
IGF-1 (*μ*g/ml)	243.44	676.98	709.43	727.00	818.44
	±124.61^ab^	±242.58	±214.72	±186.49	±192.96
TC (mmol/L)	4.53 ± 0.61^b^	3.90 ± 0.61	4.02 ± 0.47	3.83 ± 0.51	3.67 ± 0.38
HDL-C (mmol/L)	1.26 ± 0.24	1.35 ± 0.29	1.34 ± 0.30	1.39 ± 0.31	1.49 ± 0.38
LDL-C (mmol/L)	2.76 ± 0.46^ab^	2.22 ± 0.36	2.10 ± 0.28^c^	2.04 ± 0.42	1.71 ± 0.41
TG (mmol/L)	1.34 ± 0.65^b^	1.16 ± 0.55	1.06 ± 0.52	0.92 ± 0.35	0.87 ± 0.35
ALT (U/L) #	31.24 ± 21.85^ab^	19.36 ± 9.44	17.29 ± 16.02	15.17 ± 5.60	15.39 ± 4.94
HbA1c (%)	5.68 ± 0.26	5.67 ± 0.28	5.59 ± 0.25	5.62 ± 0.34	5.58 ± 0.26
FBG (mmol/L)	5.24 ± 0.33^b^	5.62 ± 0.38	5.65 ± 0.52	5.41 ± 0.48	5.61 ± 0.51
Insulin (*μ*IU/mL)#	24.34 ± 13.52	32.39 ± 13.07	29.06 ± 15.09	24.51 ± 11.51	23.66 ± 12.41
HOMA-IR #	5.67 ± 3.12	8.15 ± 3.60	7.39 ± 4.16	6.02 ± 3.06	5.97 ± 3.12

#*P* value reported for log-transformed values, but values in the table represent a back transformation. ^a^*P* < 0.05 when GH treatment of 6 months was compared with baseline. ^b^*P* < 0.05 when GH treatment of 1 year was compared with baseline. ^c^*P* < 0.05 when GH treatment of 1 year was compared with GH treatment of 6 months.

## Data Availability

All data used in this study belong to the Second Hospital of Shandong University. Data can be obtained from the corresponding author upon request.

## Abbreviations

GH: Growth hormone
IGF-1: Insulin-like growth factor 1
NAFLD: Nonalcoholic fatty liver disease
rhGH: Recombinant human growth hormone
BMI SDS: Body mass index standard deviation scores
BMI: Body mass index
Ht SDS: Height standard deviation scores
ALT: Alanine aminotransferase
TC: Total cholesterol
HDL-C: High-density lipoprotein cholesterol
LDL-C: Low-density lipoprotein cholesterol
TG: Triglycerides
FBG: Fasting blood glucose
HbA1C: Glycosylated hemoglobin
OGTT: Oral glucose tolerance test
HOMA-IR: Homeostasis model assessment-insulin resistance.
